# Functional Brain Network Analysis of Knowledge Transfer While Engineering Problem-Solving

**DOI:** 10.3389/fnhum.2021.713692

**Published:** 2021-10-25

**Authors:** Fuhua Wang, Zuhua Jiang, Xinyu Li, Lingguo Bu, Yongjun Ji

**Affiliations:** ^1^Department of Industrial Engineering and Management, Shanghai Jiao Tong University, Shanghai, China; ^2^College of Mechanical Engineering, Donghua University, Shanghai, China; ^3^School of Mechanical and Aerospace Engineering, Nanyang Technological University, Singapore, Singapore; ^4^Joint SDU-NTU Centre for Artificial Intelligence Research (C-FAIR), Shandong University, Jinan, China; ^5^School of Software, Shandong University, Jinan, China

**Keywords:** knowledge transfer, functional connectivity, brain network, wavelet phase coherence, functional near-infrared spectroscopy, cognitive structure

## Abstract

As a complex cognitive activity, knowledge transfer is mostly correlated to cognitive processes such as working memory, behavior control, and decision-making in the human brain while engineering problem-solving. It is crucial to explain how the alteration of the functional brain network occurs and how to express it, which causes the alteration of the cognitive structure of knowledge transfer. However, the neurophysiological mechanisms of knowledge transfer are rarely considered in existing studies. Thus, this study proposed functional connectivity (FC) to describe and evaluate the dynamic brain network of knowledge transfer while engineering problem-solving. In this study, we adopted the modified Wisconsin Card-Sorting Test (M-WCST) reported in the literature. The neural activation of the prefrontal cortex was continuously recorded for 31 participants using functional near-infrared spectroscopy (fNIRS). Concretely, we discussed the prior cognitive level, knowledge transfer distance, and transfer performance impacting the wavelet amplitude and wavelet phase coherence. The paired *t*-test results showed that the prior cognitive level and transfer distance significantly impact FC. The Pearson correlation coefficient showed that both wavelet amplitude and phase coherence are significantly correlated to the cognitive function of the prefrontal cortex. Therefore, brain FC is an available method to evaluate cognitive structure alteration in knowledge transfer. We also discussed why the dorsolateral prefrontal cortex (DLPFC) and occipital face area (OFA) distinguish themselves from the other brain areas in the M-WCST experiment. As an exploratory study in NeuroManagement, these findings may provide neurophysiological evidence about the functional brain network of knowledge transfer while engineering problem-solving.

## Introduction

Simulating the thinking pattern of the human brain is an attainable way to break through the bottleneck of artificial intelligence (Mohammad and Ali, 2020; Naghshvarianjahromi et al., 2020; Bin et al., 2021). Transfer learning, inspired by knowledge transfer, is a paradigm in machine learning to recognize and apply knowledge/skills learned in previous domains to the novel but related domains (Zhuang et al., 2021). Annotated data or knowledge frameworks were transferred from related fields to specific target fields or tasks situation. The rapid development of transfer learning is dependent on the exploration of knowledge transfer (Khan et al., 2019). Knowledge transfer is considered one of the most effective strategies for enterprises in knowledge-based theory. It has received considerable attention from academics and corporate governance, aiming to research what common knowledge can be transferred between different domains or tasks (Prescott et al., 2014). However, limited literature focused on the cognitive structure and brain network of knowledge transfer. The ambiguity of the neurophysiological substrates of cognitive processing cause the poor innovation performance of enterprises (Jian et al., 2019; Sungsoo et al., 2019).

*Knowledge transfer is the process whereby individual skills/knowledge in one situation impacts the skills/knowledge in another situation* (Ishizaka et al., 2020; Li et al., 2021a). Individual knowledge transfer builds the cognitive structure learned on prior experiences, not involving intra-organizational and inter-organizational interactions (Sadita et al., 2020). Engineers constantly review previous knowledge and learn new skills while engineering problem-solving, usually accompanied by multiple knowledge transfers (John et al., 2019; Vieira et al., 2020). The efficiency of knowledge transfer usually depends on the individual cognitive level when engineers solve specific problems (Li et al., 2017, 2021b). The cognitive level is concerned with the mastery of knowledge, which is a concept in cognitive psychology based on the cognitive structure of the human brain. The cognitive level is influenced by three factors: the complexity of the knowledge contained in the learning materials, the organization rules or presentation methods of the learning materials, and the experience of the learner (Macpherson and Stanovich, 2007). Bloom's Taxonomy of Cognitive Development identified six levels within the cognitive structure, i.e., knowledge, comprehension, application, analysis, synthesis, and evaluation. The cognitive level starts from the simple recall or recognition of facts, through increasingly more complex and abstract mental levels, to the highest order classified as evaluation (Rahbarnia et al., 2014). Learners with high cognitive levels could more rapidly utilize prior experience when facing a new situation, optimizing their learning footwork and elevating the learning efficiency of new knowledge (Sitzmann and Ely, 2011; Li et al., 2018). Generating and transforming representations in design ideation plays an indispensable role in engineering problem-solving (Galati and Bigliardi, 2019). Knowledge transfer enhances the conceptualization of knowledge and consequently generates critical thoughts and advanced design ideas (Spuzic et al., 2016).

*The cognitive factors of knowledge transfer are mainly comprised of the prior cognitive level, transfer distance, and transfer performance* (Galati and Bigliardi, 2019; Zhuang et al., 2021). Prior cognitive level, a series of theories or methods that individuals have acquired in their past engineering experience before solving the current technological problems, is an indicator in evaluating the validity and integrity of prior cognitive structures (Wang et al., 2021). Referring to a similar scenario in information retrieval (IR), the prior cognitive level was quantified by precision (the right predicted positive value in predicted positive examples) and recall (the right predicted positive value in all actual positive examples) (Hu et al., 2017; Li et al., 2019). Transfer distance is divided into near and far transfers, indicating the difficulty of knowledge transfer while engineering problem-solving (Sapuarachchi, 2021). Near transfer refers to the similar cognitive structure between the target and prior domains, while the far transfer is the opposite (Brunyé et al., 2020). The nature of near knowledge transfer is the recall and reuse of similar problem features, similar application contexts, and similar knowledge elements. With opposite connotations during far transfer, individuals draw on dissimilar knowledge learned in previous scenarios to solve current engineering problems. The manifestations of the far transfer problems are pretty distinct from prior experience, but their knowledge elements are relevant in the deep structure of knowledge. The WordNet-based semantic similarity in natural language processing has been used to measure the similarity of the two cognitive structures and to quantify the transfer distance of empirical engineering knowledge under different technological paradigm shifts (Wang et al., 2021). Transfer performance evaluated the ability of engineers to solve new technological problems after knowledge transfer (Wang et al., 2018). The validity, integrity, and availability of the cognitive structure dominate the process and performance of knowledge transfer. Questionnaires and scales assessed the performance of knowledge transfer, such as Montreal Cognitive Assessment (MoCA), Mini-Mental State Exam (MMSE), or Cognitive Reserve Scale (CRS) (Bu et al., 2018a).

The brain is a computational and decision-making system that contains complex networks. Executing a particular cognitive activity requires synergizing several functional brain regions and sophisticated functional networks consist of these connectivities in the human brain system (Bu et al., 2020b). Knowledge transfer, a complicated cognitive activity, is usually correlated to cognitive processes such as working memory, control, and decision-making in the human brain. It is crucial to explain how the alteration of the functional brain network occurs and how to express it, which causes the alteration of the cognitive structure of knowledge transfer (Kulasegaram et al., 2017). With the development of cognitive science and neuroimaging technology, functional connectivity (FC) analysis provides an executable method to reveal the neural activity and connectivity of brain function (Bu et al., 2018a). Resting-state functional connectivity (rsFC) indicates the flow of information among brain regions and measures the strength of its connection (Bu et al., 2018b; Tozzi et al., 2021). Related works have used wavelet phase coherence (WPCO) to characterize the rsFC between different brain regions. The WPCO assessed the impaired cognitive function of subjects with hypertension (Brunyé et al., 2020). The WPCO evaluated dynamic cerebral autoregulation in neonatal hypoxic-ischemic encephalopathy (Zhang et al., 2020). Analyzing the functional connectivity of knowledge transfer allows an in-depth detection of the neurovascular coupling mechanisms while engineering problem-solving. Related works found rsFC in the prefrontal cortex (PFC), whether in the resting state or task state (Antzoulatos and Miller, 2014). The reasons focusing on rsFC in this study are as follows. First, there are few studies on task-state FC (Cole et al., 2021), but presently, the rsFC method has been a thorough and popular analysis method on brain networks. Second, task-state and resting-state FC use completely different algorithms. Hence, the research mode is different. Some classical experimental paradigms can adopt task-state FC because their stimuli are generic and regular. Nevertheless, due to the exceedingly complex knowledge transfer process, the existing research cannot be studied through classical technology paradigms, complicating the task-state FC (Zhang et al., 2020). As far as the current researches on the neural substrates of knowledge transfer are concerned, it is difficult to analyze and explain the causes/mechanisms of the generation/defect of task-state FC in the knowledge transfer process.

In recent years, functional near-infrared spectroscopy (fNIRS) has been widely used in FC in complex cognitive processing (Arun et al., 2020; Bu et al., 2021). Grounded on the optical properties of biological tissues and the modified Beer-Lambert law, fNIRS acquires the biochemical information in 650–900 nm light after a series of absorption and scattering (Bu et al., 2020a). Oxyhemoglobin (HbO) and deoxyhemoglobin (HbR) quantify the concentration of chromophores. It provides convenient and authentic monitoring indicators for clinical research by collecting fNIRS data related to tissue and physiological function (Bu et al., 2019; Nakamura et al., 2020). Compared with functional magnetic resonance imaging (fMRI) and electroencephalogram (EEG), fNIRS can provide a non-destructive brain region detection and a high temporal and spatial resolution. fNIRS is also portable, low constraints, and longtime repeated measurement. Hence it has gotten more attention in cognitive neuropsychology (Bu et al., 2017; Udina et al., 2020).

The existing studies on knowledge transfer were widely researched in pedagogy (Bae et al., 2019; Lombardi, 2019), management (de Wit-de Vries et al., 2019; Li et al., 2020), and information science (Bacon et al., 2019; Sun et al., 2020). However, the literature mainly focused on bibliometrics analysis (Prihodova et al., 2019) and behavioral measurement (Marques et al., 2019). Their conclusions were affected by the subjective willingness of subjects, resulting in the reduction of interpretability (Vasudev and Pooja, 2020; Xie et al., 2020). Moreover, the related works did not involve how the dynamic alteration of the functional brain network occurs and how to reveal it. Modified Wisconsin Card-Sorting Test (M-WCST) and FC analysis provide feasibility for quantifying the alteration. The WCST is a neuropsychological test, which was put forward to assess abstract thinking and learning ability (Grant and Berg, 1948). This neuropsychological method is sensitive in the PFC especially the dorsolateral prefrontal cortex (DLPFC) (Shirayama et al., 2010; Steinke et al., 2021). It measures the cognitive capability of abstract generalization, material extraction, working memory, and knowledge transfer by utilizing prior experience. The test was modified by different scholars given the different experimental needs (Kopp et al., 2019; Gómez-de-Regil, 2020). This study adopts a proven feasible M-WCST to evaluate the concept classification ability of subjects after knowledge transfer (Wang et al., 2021).

This study is a follow-up to the study of Wang et al. (2021). In the mentioned study, the significantly activated brain regions in knowledge transfer were revealed by statistical parametric mapping (SPM treatment), which was an initial but essential step for researching the neural underpinnings of knowledge transfer by fNIRS. However, the brain is a computational and decision-making system that contains complex networks. The SPM treatment has limitations because this method isolates the connection between the brain regions. It cannot analyze or reveal the synchronization and synergy of networks among the brain regions.

This study proposes neurophysiological evidence about the brain network underlying rsFC, thus is one step further in promoting knowledge transfer research in engineering problem-solving. The cognitive level of knowledge transfer from brain activity was analyzed in different situations (near transfer and far transfer). We discussed the prior cognitive level and transfer distance affecting functional brain connectivity in knowledge transfer. Wavelet amplitude and WPCO were applied to evaluate the FC. Transfer performance analysis showed that the FC trustworthily assessed the cognitive level of subjects. The three groups of hypotheses are as follows:

**H1**: The distance of knowledge transfer is significantly correlated to FC;

- **H1a**: The distance of knowledge transfer is significantly correlated to the wavelet amplitude of FC;
- **H1b**: The distance of knowledge transfer is significantly correlated to the WPCO of FC;

**H2**: Prior cognitive level is significantly correlated to FC;

- **H2a**: Prior cognitive level is significantly correlated to the wavelet amplitude of FC;
- **H2b**: Prior cognitive level is significantly correlated to the WPCO of FC;

**H3**: The transfer performance is significantly correlated to FC.

- **H3a**: The transfer performance is significantly correlated to the wavelet amplitude of FC;
- **H3b**: The transfer performance is significantly correlated to the WPCO of FC;

## Materials and Methods

### Subjects

A total of 33 subjects were recruited at the School of Mechanical Engineering, Shanghai Jiao Tong University, China. The experiment was finished by 31 subjects (age: 21.0526 ± 1.8995) (excluding two participants, who did not follow the requirements), of which 24 are males and 7 are females. All participants satisfied the following criteria: (1) no structural abnormalities, (2) no neurological or psychiatric disorders, (3) no use of medication, (4) right-handed, and (5) healthy vision. Before the experiment, all participants signed an informed consent approved by the Institutional Review Board (IRB) of the Shanghai Jiao Tong University. It was in accordance with the ethical standards specified by the Helsinki Declaration of 1975 (revised in 2008). Anonymous information on the subjects was recorded before the test, such as gender and age.

### Experimental Measurements

The M-WCST experiment assessed the functional brain network of knowledge transfer while engineering problem-solving. It was divided into three phases, namely, prior technological paradigms (TP), near-transferred TP, and far-transferred TP, each phase taking about 30 min. In each phase, the subjects needed to solve specific design problems about three engineering products. The engineering products are as follows: a cylindrical speed-reducer gearbox (prior TP), a planetary speed-increaser gearbox (near-transferred TP), and an involute gear pump (far-transferred TP) (Wang et al., 2021). The stimulus materials were extracted from the glossary of terms in the authoritative monographs of the *Handbook of Machine Design* and *Hydrodynamics and Hydraulic Transmission*. Therefore, all the stimulus materials exhibited on the screen were knowledge concepts about engineering design, and all the concept-sorting tests were the specific problems to be solved for the subjects.

The cylindrical speed-reducer gearbox is a traditional and representative transmission mechanism. Most students who major in mechanical design or designers whose occupations are mechanical design are familiar with this gearbox design. The TP covers the commonly used and basic concepts. Therefore, it can measure the cognitive level of the subject with regards to the prior TP. The planetary speed-increaser gearbox with a multi-stage epicyclic gear train increases the difficulty and complexity of the design task. Compared with the cylindrical speed-reducer gearbox, the planetary speed-increaser gearbox increases the similarity and availability of knowledge, but it still belongs to the category of gear transmission. Hence, it can measure the cognitive level of the near-transferred TP. Compared with the two mechanisms above, designing an involute gear pump requires basic concepts of gears transmission and even the knowledge of hydrodynamics and hydraulic transmission. Hence, the low similarity increases the difficulty of knowledge transfer. Although the two products are different, the common element, “external gear,” is related to the deep structure of knowledge. Therefore, it can measure the far transfer performance.

It can be seen above that the experiment consists of three different TPs depended on the distance of knowledge transfer. Furthermore, several experienced domain experts divided engineering concepts into three groups through the Delphi method and the WordNet-based semantic similarity. These specific concepts were exhibited to subjects in each design stage of the M-WCST experiment. Half of them are related/unrelated concepts to each design problem in each stage.

**Figure 1:**
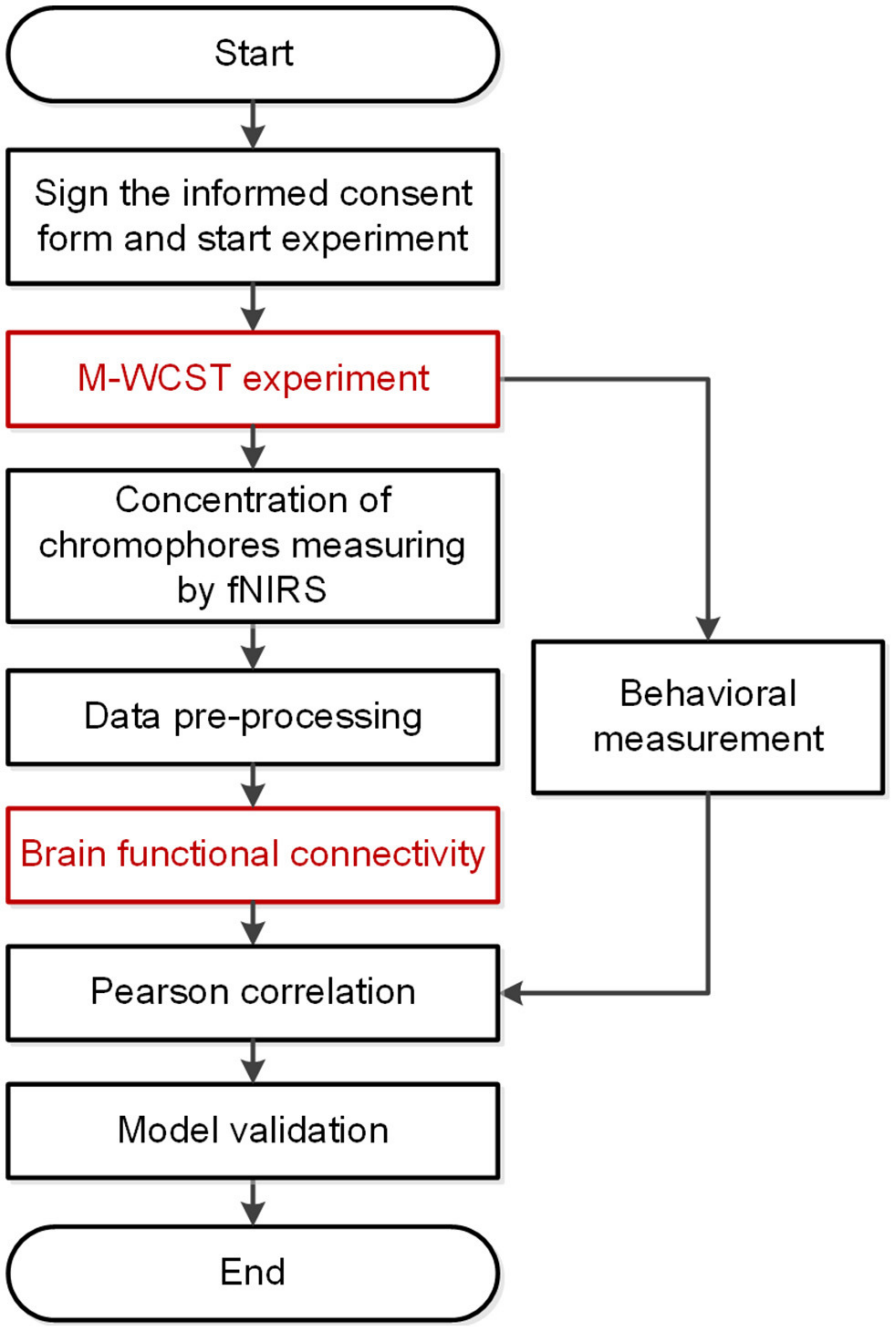
Flowchart of the functional brain network analysis of knowledge transfer.

The flowchart of the experiment is shown in Figure 1. In the M-WCST experiment, the ΔHbO concentration in the brain area was measured by fNIRS. The prior cognitive level, transfer distance, and transfer performance were tested using the concept-sorting tests, which were proposed in previous research (Wang et al., 2021). Then the two parameters of wavelet amplitude and phase coherence were calculated after pre-processing and wavelet transform. The significant correlations of prior cognitive level and transfer distance on functional connectivity were analyzed by a paired-samples *t*-test. Through Pearson correlation coefficient, we confirmed the feasibility of FC to explore the neurophysiological substrates of knowledge transfer.

**Figure 2:**
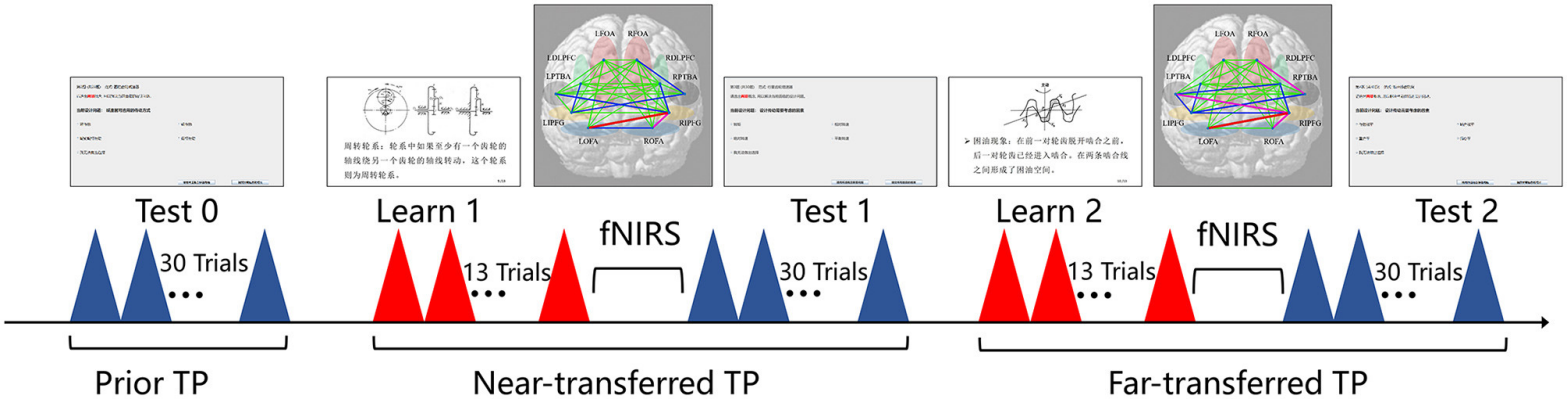
Stimulus sequences and stimulus materials.

As shown in Figure 2, in the prior TP (design task of the cylindrical speed-reducer gearbox), the subjects finished the concept-sorting test under the prior TP to collect the prior cognitive level. In the near-transferred TP (design task of the planetary speed-increaser gearbox), they learned new knowledge from the near knowledge transfer. Then they kept their resting state after the learning task for about 10 min, and we collected the ΔHbO concentration using fNIRS during this time. Afterward, they finished a concept-sorting test to collect the performance of near knowledge transfer. In the far-transferred TP (design task of involute gear pump), they learned new knowledge from the far knowledge transfer, and then the ΔHbO concentration of resting-state after learning task was collected. Afterward, the performance under the far-transferred technological paradigm was collected. The exhibiting order of the near-transferred and far-transferred TP was random.

**Figure 3:**
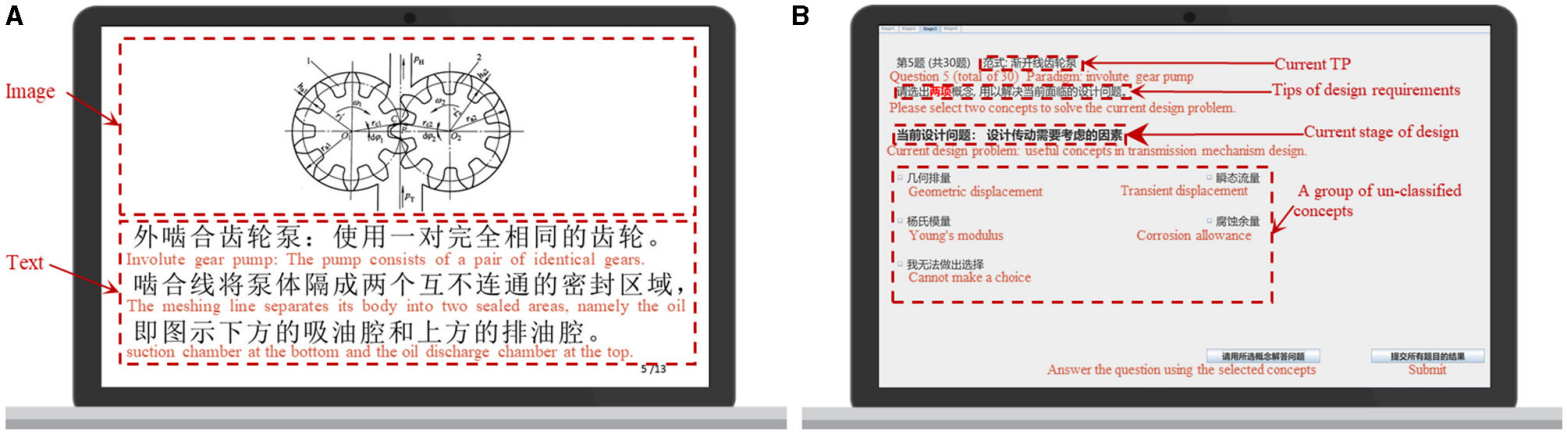
Stimulus materials we provided for the subjects. **(A)** Learning tasks; **(B)** concept-sorting tests.

In the learning tasks, the subjects watched 13 trials of stimulus materials. As shown in Figure 3A, the stimulus materials we provided for the subjects are the learning contents in the new technology paradigm, including text or image. Each stimulus had a different duration, such as 5 or 30 s. The two learning tasks lasted for 5 min.

In the concept-sorting tests, as shown in Figure 3B, first, we introduced the research purpose, operation procedure, and precautions to the subjects. Second, they read the scenario to comprehend the objects, parameters, and constraints of the current design task. Finally, they finished 30 concept classification trials independently and submitted all the results in the formal experiment. According to the discernibility of their cognitive structure, the subjects selected the concepts solving current design problems. Therefore, those concepts were divided into two categories, “related to design intention” and “unrelated to design intention.” The human-computer interaction system recorded a timestamp in the background (synchronously marking the fNIRS data) then took a 10 s break. The three concept-sorting tests lasted for about 25 min.

### Experimental Data Acquisition and Processing

A multichannel fNIRS device measured the blood oxygen level-dependent (BOLD). After the data pre-processing, the FC was calculated using the wavelet transform (wavelet amplitude and phase coherence). Pearson correlation analysis sustained the relevance between transfer performance and functional connectivity.

### Data Acquisition

**Figure 4:**
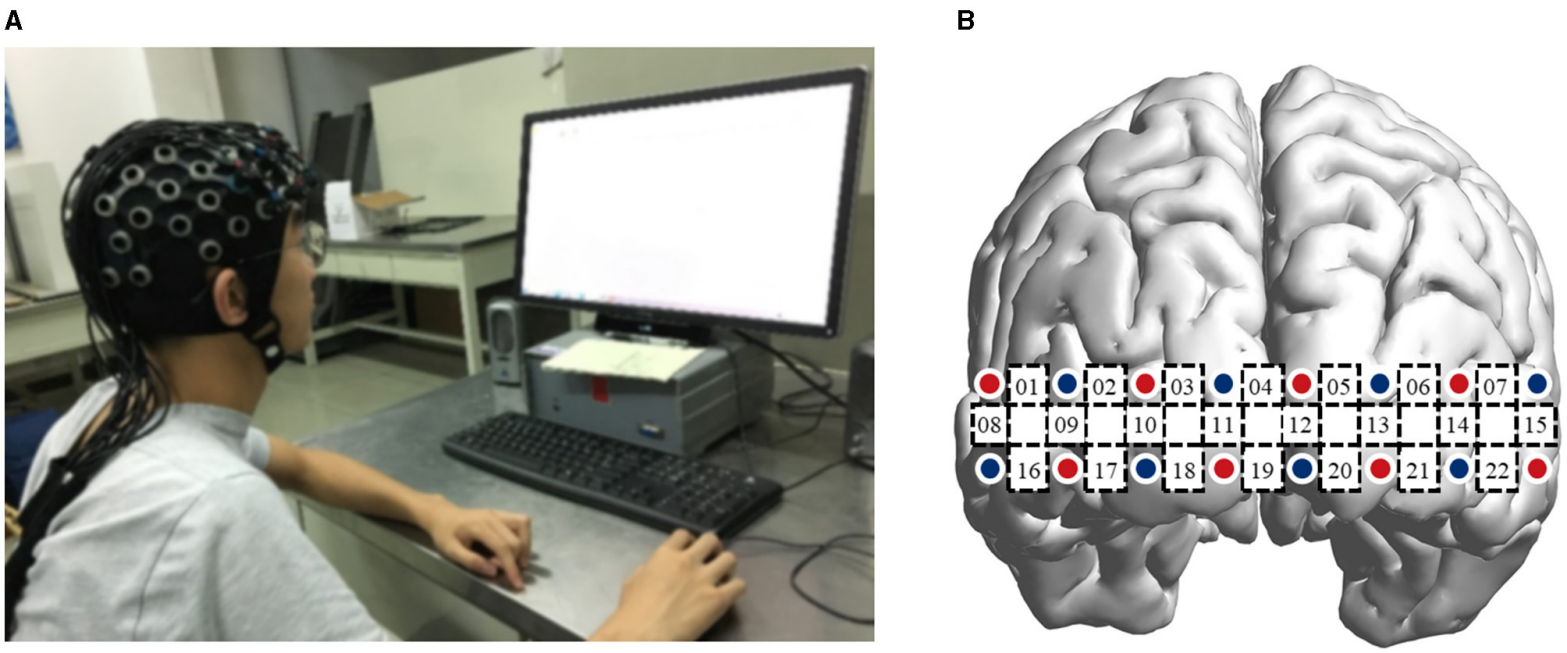
Experimental environment and channel layout. **(A)** The experimental situation of subjects; **(B)** configuration of optode emitters (red), detectors (blue), and channels (yellow).

The experiment of knowledge transfer was conducted in an enclosed and quiet laboratory environment, as shown in Figure 4A. A Shimadzu LIGHTNIRS device (Shimadzu Corporation, Nishinokyo Kuwabara-cho, Nakagyo-ku, Kyoto 604-8511, Japan) was used to detect ΔHbO and ΔHbR concentrations in the PFC at the sampling rate of 13.33 Hz. The three wavelengths of near-infrared light were 780, 805, and 830 nm. The 22 channels (CHs) comprised 8 × 2 optode probes (8 emitters and 8 detectors), which covered the PFC. The exact positions of the optode emitters and detectors are shown in Figure 4B, following the standard international 10/20 system. A three-dimensional (3D) digitizer determined the spatial registration of the fNIRS channels on the MNI space.

(Shimadzu Corporation, Nishinokyo Kuwabara-cho, Nakagyo-ku, Kyoto 604-8511, Japan)

The brain regions covered by fNIRS are shown in Table 1, also the regions of interest (ROIs) in this experiment.

**Table 1:** ROI-channel mapping.

**#**	**Brain regions**	**CHs**
10	FOA (frontopolar area)	L: CH02, CH03, CH04, CH10, CH11
		R: CH04, CH05, CH06, CH12, CH13
11	OFA (orbitofrontal area)	L: CH18, CH19
		R: CH19, CH20
45	PTBA (pars triangularis Broca's area)	L: CH08
		R: CH15
46	DLPFC (dorsolateral prefrontal cortex)	L: CH01, CH09, CH17
		R: CH07, CH14, CH21
47	IPFG (inferior prefrontal gyrus)	L: CH16
		R: CH22

### Data Pre-processing

The pre-processing of the cerebral oxygen signal will eliminate outliers and increase the signal-to-noise ratio so that the overall filtered signal is convenient for subsequent calculation and analysis. First, like all experimental instruments or equipment, the fNIRS equipment chose in this experiment (Shimadzu LIGHTNIRS) also has high-frequency noise during data acquisition, such as system noise and ambient light interference. Second, the subject's head movement and other reasons will bring low-frequency noise to the signal. Hence signal pre-processing also aims to detrend drift noise and movement artifacts, and the filtered signal can be used as the actual fNIRS signal. In this study, the raw data acquired by fNIRS was pre-processed through the NIRS_SPM toolbox of MATLAB R2013b, which included:

(1) registration of MNI coordinates;
(2) construction of the design matrix based on General Linear Model (GLM);
(3) low-pass filter based on hemodynamic response function (HRF) with time derivative;
(4) high-pass filter based on Discrete Cosine Transform (DCT) detrending algorithm.

Afterward, SPSS 22.0 software supports statistical analysis and hypothesis tests to behavioral and pre-processed data.

### Frequency Intervals of Blood Oxygen Signal

The different frequency intervals of blood oscillation signify different physiological connotations, specifically in the frequency intervals (0.0095–2.0 Hz). To extract the target signals from the filtered fNIRS signals, the spontaneous blood oscillation signal was divided into six frequency intervals (Shiogai et al., 2010): I, 0.6–2 Hz, cardiac activity; II, 0.145–0.6 Hz, respiration; III, 0.052–0.145 Hz, myogenic activity; IV, 0.021–0.052 Hz, neurogenic activity; V, 0.0095–0.021 Hz, endothelial metabolic activity; and VI, 0.0005–0.0095 Hz, endothelial activity.

In this study, the inherent frequency of the neurogenic activity of the PFC in the resting state was 0.021–0.052 Hz; on the other hand, the stimulus frequency of the experiment was about 0.014–0.085 Hz for the learning phase and 0.011–0.056 Hz for the testing phase. Combining the frequency of the neurogenic activity and stimulus exhibition, we focused on the 0.01–0.1 Hz frequency intervals correlated to the functional connectivity of knowledge transfer while engineering problem-solving.

### Functional Connectivity

Functional connectivity refers to the dynamic synchronization of neural signals, revealing synergies among different brain regions. Since the functional brain network is dynamically altering, functional connectivity requires time-frequency signal processing methods. In previous studies, WPCO has been extensively used for interactive FC analysis, elaborating the phase correlation between the two blood oxygen signals (Bu et al., 2018b). The WPCO can appraise neural activation in different regions that are independent of amplitude. This study adopted WPCO to calculate the phase synchronization value. The calculation process of wavelet amplitude and phase coherence are briefly described below.

#### Wavelet Amplitude

Previous studies reported the wavelet transform method in detail, transforming non-stationary blood oxygen signals from the time domain to the time-frequency domain (Bandrivskyy et al., 2004). It allows time-varying signals to decompose amplitude and phase with the time-frequency cardinal (Arun et al., 2020). For a discrete signal *x*(ω_*k*_, *t*_*n*_) at a certain frequency ω_*k*_ ∈ {*I, VI*} and a certain moment *t*_*n*_ ∈ (0, *T*) from different channels measured by fNIRS, we can get its complex spectral function through wavelet transform.

Many types of wavelet basis functions are available, such as the Haar, Daubechies, Gaussian, Meyer, Mexican Hat, Morlet, Coiflet, Symlet, and Biorthogonal. There is no golden standard to determine which wavelet function will be appropriate for a specific task. In actuality, the type of wavelet is determined according to the characteristics of the collected signals. In this study, the Morlet wavelet we selected in this experiment, mainly referring to related studies (Bu et al., 2018b; Mahadevan et al., 2021). Following the related studies, the non-orthogonal basis function we chose in this experiment is the complex Morlet wavelet to decompose the fNIRS signal to calculate the WPCO. Investigating the existing research, we also used the Morlet wavelet in the data processing. The bandwidth of 2 and center frequency of 0.5 used in this study are the empirical values obtained through the debugging of the MATLAB program (The MathWorks, Inc., Natick, Massachusetts, USA). As a result, the data processing using Morlet achieves a positive effect and meets the needs of the fNIRS signal decomposition in this study.

The wavelet coefficients are complex in the time-frequency domain.


(1) $$\begin{array}{l} x\left(\omega_{k},t_{n}\right)=|X_{k}|e^{i\phi_{k,n}}=a_{k,n}+ib_{k,n} \end{array}$$

The wavelet phrase ϕ_*k, n*_ is defined as


(2) $$\begin{array}{l} \phi_{k,n}=arctan\left(\frac{b_{k,n}}{a_{k,n}}\right) \end{array}$$

The wavelet amplitude |*x*_*k*_| at ω_*k*_ averaged in the time domain for the whole time series is defined as


(3) $$\begin{array}{l} |x_{k}|=\frac{1}{T}\overset{T}{\underset{n=0}{\sum }}\sqrt{a_{k,n}^{2}+b_{k,n}^{2}} \end{array}$$

After wavelet transform, we obtained the amplitude-frequency correlation.

#### Wavelet Phase Coherence

The amplitude-independent WPCO is assessed in this section (Lees et al., 2021). For the two discrete signals from two different fNIRS channels, wavelet-transformed to the time series *x*_*i*_(ω_*k*_, *t*_*n*_) and *x*_*j*_(ω_*k*_, *t*_*n*_), one gets two corresponding instantaneous phases ϕ_*i*_(ω_*k*_, *t*_*n*_) and ϕ_*j*_(ω_*k*_, *t*_*n*_) in Equation 2. Then, the relative phase difference Δϕ_*k, n*_ = ϕ_*j*_(ω_*k*_, *t*_*n*_) − ϕ_*i*_(ω_*k*_, *t*_*n*_) can be computed. Subsequently, the trigonometric coefficients cosΔϕ_*k, n*_ and sinΔϕ_*k, n*_ are averaged in the time domain for the whole time series. Finally, the time-averaged WPCO was defined as


(4) $$\begin{array}{l} WPCO\left(\omega_{k}\right)=\sqrt{{\left(\frac{1}{T}\overset{T}{\underset{n=0}{\sum }}\cos\Delta \phi_{k,n}\right)}^{2}+{\left(\frac{1}{T}\overset{T}{\underset{n=0}{\sum }}\sin\Delta \phi_{k,n}\right)}^{2}} \end{array}$$

0 ≤ *WPCO*(ω_*k*_) ≤ 1.*WPCO*(ω_*k*_) can assess the strength of phase coherence between two signals in different brain regions. If the coherence coefficient is close to zero, then, the two regions are uncorrelated. *WPCO*(ω_*k*_) = 1 would correspond to complete coherence between the two regions.

However, *WPCO*(ω_*k*_) does not accurately indicate the synchronization of the two signals since it is easily disturbed by frequency. Concretely, in a signal with finite length, the low-frequency component can be represented by fewer periods than the high-frequency component (Antzoulatos and Miller, 2014). Hence, the phase difference of the signal has a minor variation at low frequency, which artificially increases the phase coherence of the low-frequency component. Therefore, the coefficient of the phase coherence increases monotonically as the frequency decreases, even though the two signals are entirely asynchronous (Marusak et al., 2017).

**Figure 5:**
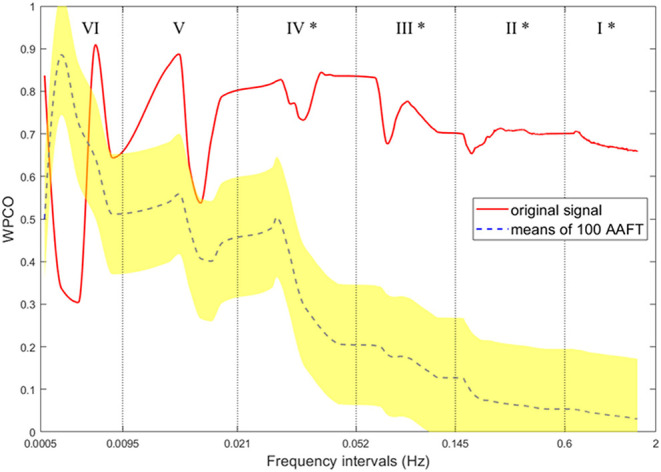
Wavelet phase coherence. Original (red) and Alternative (blue) signals, subject #12, channel #01, channel #02.

To ensure the consistency between the WPCO and the synchronization of two signals, the amplitude adjusted Fourier-transform (AAFT) method was used to produce alternative signals to verify the coherence coefficients values (Tóth et al., 2017). Specifically, 100 alternative signals were generated from the original signal by the AAFT transform. Suppose the WPCO of the original signal is higher than the sum of the mean and twice the SD of the WPCO of the alternative signals. In that case, it indicates significant coherence between the two channels of the original signal, and the value of its WPCO is valid. *valid*(ω_*k*_) is defined as


(5) $$\begin{array}{l} valid\left(\omega_{k}\right)=WPCO_{origin}\left(\omega_{k}\right)-\\ \left(\frac{1}{100}\overset{100}{\underset{m=1}{\sum }}WPCO_{m}^{AAFT}\left(\omega_{k}\right)+2{\;}^{*}\;td\left(WPCO_{m}^{AAFT}\left(\omega_{k}\right)\right)\right)>0, \end{array}$$

whenever ω_*k*_ ∈ (0, *f*_*I*~*VI*_,). The coherence curves (original and alternative signals) from one subject are shown in Figure 5. Therefore, the whole calculating procedure of the FC is as follows: (1) wavelet transforming the signals of each channel; (2) calculating the *WPCO*(ω_*k*_) between the two channels separately; (3) generating alternative signals using the AAFT method, and checking the *valid*(ω_*k*_) of WPCO in Equation (5).

#### Functional Brain Network Analysis

**Figure 6:**
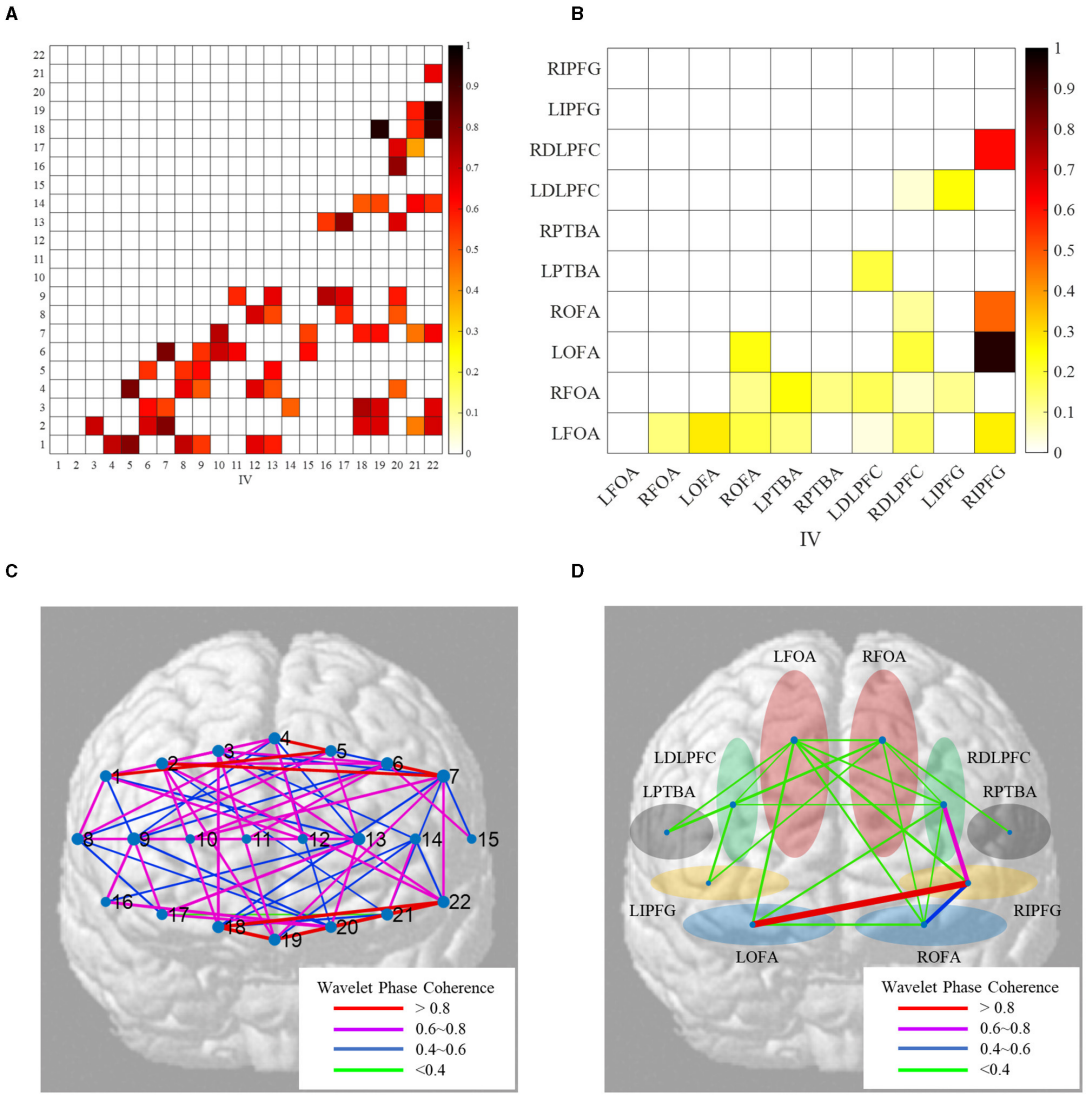
Heatmaps of wavelet phase coherence and functional brain network of ROIs (subject #12). **(A)** Heatmaps of WPCO between channels; **(B)** heatmaps of WPCO between ROIs; **(C)** map of the functional brain network between channels; **(D)** map of the functional brain network between ROIs.

As mentioned above, we obtained the valid coefficients of WPCO between the two channels. If *valid*(ω_*k*_) > 0, the WPCO of the brain regions is the mean of the WPCO of the related channels. Contrarily, if *valid*(ω_*k*_) ≤ 0, the WPCO of the brain regions is 0. The heatmaps of wavelet phase coherence and functional brain network of brain regions (subject #12) are shown in Figure 6.

In Figures 6A,B, not all WPCO between the two channels are valid due to the missing connectivity. The heatmaps show that the FC is sparse. Through the ROI-Channel mapping shown in Table 1, we can average the WPCO values of the channels on the same region and then obtain the brain network in Figures 6C,D. The map of the functional brain network intuitively shows the dynamic synchronization of neural signals and reveals synergy between brain regions.

## Results

**Figure 7:**
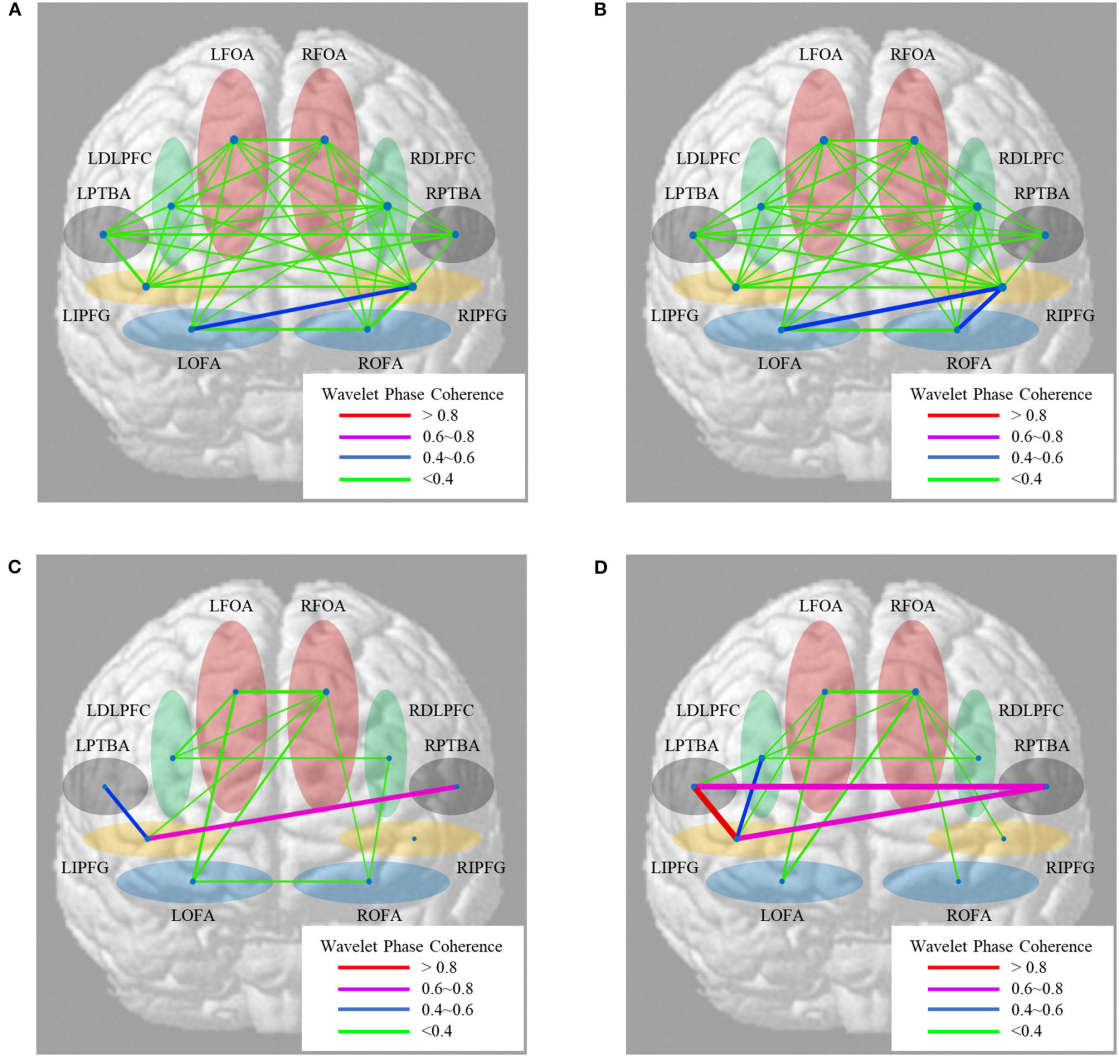
Maps of the functional brain network between brain regions averaging over all subjects. **(A)** Near-transferred group; **(B)** far-transferred group; **(C)** low prior cognitive level group in far transfer; **(D)** high prior cognitive level group in far transfer.

**Figure 8:**
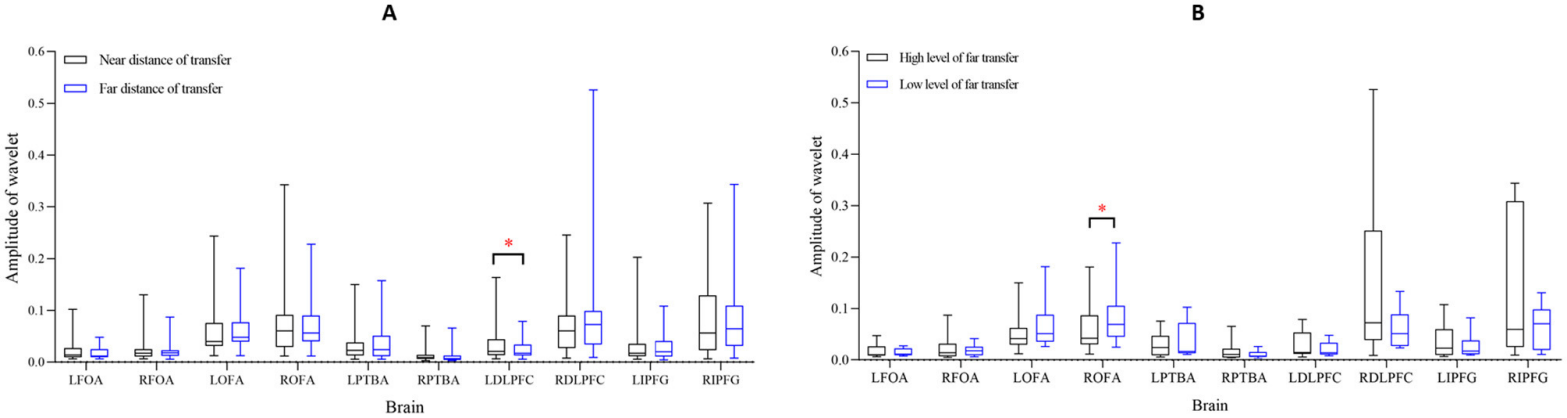
Box diagram of significant differences in wavelet amplitude. **(A)** At different knowledge transfer distances; **(B)** at different prior cognitive levels. ***Sig. < 0.001; **Sig. < 0.01; *Sig. < 0.05.

**Figure 9:**
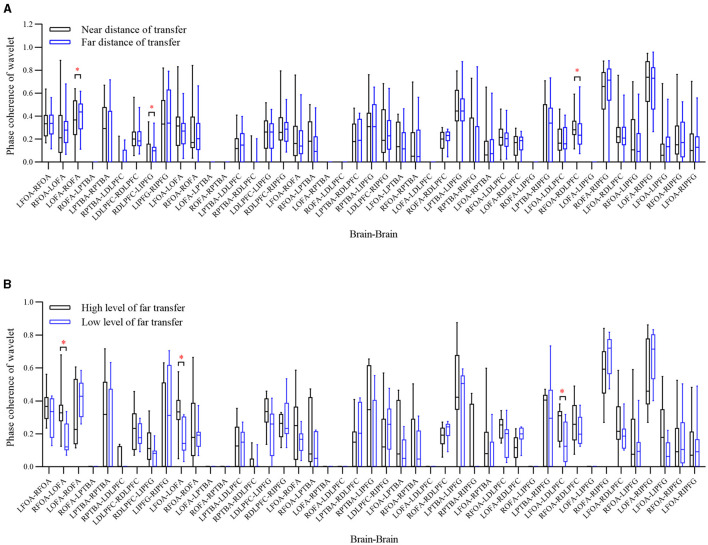
Box diagram of significant differences in wavelet amplitude. **(A)** At different knowledge transfer distances; **(B)** at different prior cognitive levels. ***Sig. < 0.001; **Sig. < 0.01; *Sig. < 0.05.

The five functional regions are divided into 10 ROIs in the left and right brain. As shown in Figure 7, the functional brain network consists of mean WPCO over all subjects. The wavelet amplitude between brain regions averaging over all subjects is shown in Figure 8. The significant activation of connectivities between these ROIs is shown in Figure 9.

### Different Distances of Knowledge Transfer

According to the distance of knowledge transfer in M-WCST, we analyzed the wavelet amplitude and WPCO of blood oxygen signals in the near-transferred TP and far-transferred TP. For all subjects, the functional brain network of near transfer is shown in Figure 7A, and the brain functional network of far transfer is shown in Figure 7B. A paired sample *t*-test was used to contrast the near-transferred TP group with far-transferred TP group. The significant differences in wavelet amplitude under different transfer distances are shown in Figure 8A. The results of the WPCO between the two brain regions under different transfer distances are shown in Figure 9A.

Comparing the near-transferred and far-transferred groups, we found significant difference in the wavelet amplitude of **LDLPFC** (*t* = 5.713, *p* = 0.0243 < 0.05, df = 30) under different transfer distances. It indicates that far transfer distances decreased the activation of the partial brain regions.

Comparing the WPCO of the two groups above, we found that **LOFA-ROFA** (*t* = 1.920, *p* = 0.0398 < 0.05, df = 30), **LIPFG-RDLPFC** (*t* = 1.991, *p* = 0.0285 < 0.05, df = 30), and **RFOA-RDLPFC** (*t* = 2.726, *p* = 0.0472 < 0.05, df = 30) were significantly different. It indicated that the transfer distance impacted the elaboration of the brain network in the PFC.

Notably, the DLPFC had significant activation under both near and far knowledge transfer. Cognitive results also illustrated a similar neurophysiological mechanism in both near and far transfer when the engineers were solving design problems since their activated brain regions partially overlap in different transfer distances.

### Different Prior Cognitive Level

According to the cognitive level in the prior TP of M-WCST, the subjects were divided into the high cognitive level (top 27%, nine people) and low cognitive level (bottom 27%, nine people). We analyzed the wavelet amplitude and WPCO in the two groups. For all the subjects, the functional brain network in the high-level group is shown in Figure 7C, and the functional brain network in the low-level group is shown in Figure 7D. The relationship between the two groups was analyzed by a paired samples *t*-test. The significant differences in wavelet amplitude under different prior cognitive levels are shown in Figure 8B. Moreover, the statistical results of the WPCO between the two brain regions under different prior cognitive levels are shown in Figure 9B.

Comparison between the high-level and low-level groups showed a significant difference in the wavelet amplitudes of **ROFA** (*t* = 6.141, *p* = 0.0421 < 0.05, df = 8) under different prior cognitive levels. It indicated that the two groups with different prior cognitive levels had different activation in brain areas. While facing some complicated problems in the concept-sorting test, the subjects with a high cognitive level could utilize more brain regions to learn knowledge than those with weak basic skills.

Comparing the WPCO of the two groups above, we found that **RFOA-LOFA** (*t* = 2.334, *p* = 0.0198 < 0.05, df = 8), **LFOA-LOFA** (*t* = 1.092, *p* = 0.0189 < 0.05, df = 8), **LFOA-LDLPFC** (*t* = 2.559, *p* = 0.031 < 0.05, df = 8) were significantly different. The statistical results showed that the WPCO of the functional connectivity was significantly different depending on the prior cognitive level. It indicated that subjects with high prior cognitive levels usually hold complex brain networks in the PFC while engineering problem-solving.

### Pearson Correlation Analysis Between the Performance of Knowledge Transfer and Functional Connectivity

**Figure 10:**
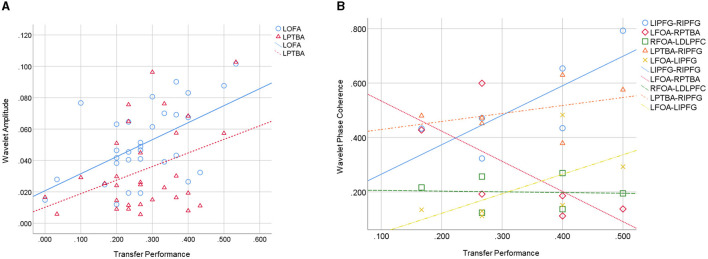
Scatter diagram of Pearson correlation analysis between transfer performance and functional connectivity. **(A)** Wavelet amplitude; **(B)** wavelet phase coherence.

To verify the effectiveness of FC and explore the neurocognitive underpinnings of knowledge transfer, the Pearson correlation coefficient analyzed the associations among wavelet amplitude, WPCO, and transfer performance. As shown in Figure 10, the scatter diagram only draws the significantly activated brain areas or connections to prevent the confusing exhibition.

The transfer performance and wavelet amplitude of **LOFA** (*r* = 0.545, *p* = 0.002 < 0.01) and **LPTBA** (*r* = 0.377, *p* = 0.036 < 0.05) showed significant positive correlation. Moreover, significant positive correlation was observed in the performance and WPCO of **LIPFG-RIPFG** (*r* = 0.623, *p* = 0.006 < 0.01), **LPTBA-RIPFG** (*r* = 0.542, *p* = 0.017 < 0.05), and **LFOA-LIPFG** (*r* = 0.564, *p* = 0.008 < 0.01). Correlation analysis also revealed that the performance was significantly negatively correlated with the WPCO of **LFOA-RPTBA** (*r* = −0.564, *p* = 0.018 < 0.05) and **RFOA-LDLPFC** (*r* = −0.369, *p* = 0.041 < 0.05). These statistical results certified that the procedure of knowledge transfer for engineering problem-solving involves the activation and inhibition of partial brain interconnectedness.

## Discussion

This section interprets the results of the wavelet amplitude and WPCO of FC in the M-WCST experiment while engineering problem-solving (near transfer and far transfer). We discuss whether the functional brain network is an efficacious and trustworthy evaluation tool to study the neurocognitive underpinnings of knowledge transfer.

### Effects of Transfer Distance on Functional Connectivity While Engineering Problem-Solving

Different Distances of Knowledge Transfer section shows that the wavelet amplitude and WPCO of FC are significantly different with the altering transfer distance. Therefore, the hypothesis **H1a** and **H1b** cannot be rejected, i.e., FC is significantly different due to different knowledge transfer distances.

Transfer distance disturbs the process of information extraction and the attention time to the effective stimulus materials of subjects, thereby affecting the brain functional connectivity (Maksimenko et al., 2019). In near-transferred TP, the current learning contents are highly similar to those in prior TP. Hence, the engineers absorbed less new knowledge, drooping the brain activation of ROI (wavelet amplitude) and increasing the complexity of the functional brain network (wavelet phase coherence). With the growing distance of knowledge transfer in far-transferred TP, there is a big gap between their current learning contents and basic skills. With the new knowledge increasing significantly, the activation of brain regions ascends, and the FC becomes intricate (Cartwright et al., 2020). It indicates that there is a significant positive correlation between transfer distance and FC.

Many previous studies substantiated that the functional brain network was a reliable method to assess neurocognitive results (Marusak et al., 2017; Lees et al., 2021). The β value of statistical parametric mapping (SPM) in related works was used to represent the cognitive load of knowledge transfer while engineering problem-solving (Wang et al., 2021). Those results indicated that a higher cognitive load means a higher significant activation of brain regions (Thees et al., 2020). The results of the wavelet amplitude in this study are consistent with the previous research. Therefore, wavelet amplitude may evaluate the cognitive load of knowledge transfer.

We analyzed the functional brain network of knowledge transfer from the perspective of knowledge networks in different TPs. Different distances of knowledge transfer denoted different TPs, which may activate the different default spontaneous activities and connectivity patterns of the brain network (Dai et al., 2015; Tóth et al., 2017; Shine, 2021). As a standard TP to mechanical design engineers, we know that the knowledge structure of the prior TP (cylindrical speed-reducer gearbox) and near-transferred TP (planetary speed-increaser gearbox) is remarkably similar. The assimilating concepts are called “knowledge transfer points” (Cartwright et al., 2020). Related works on the N-Back task showed that working memory significantly affected the near transfer effect mode of knowledge, which was superimposed by cognitive structures and learning strategies (Soveri et al., 2017). Due to the considerable tolerance to the prior TP, engineers can easily remold the initial knowledge structure and transfer it from a cylindrical speed-reducer gearbox to the new knowledge framework of reducer design. However, it is relatively complex for engineers to transfer the design knowledge from gear transmission to the hydraulic drive device for an involute gear pump. Therefore, compared with the near transfer, engineers need more brain interconnectedness to solve complex engineering problems, thus the corresponding brain network is complicated.

### Effects of Prior Cognitive Level on Functional Connectivity While Engineering Problem-Solving

From the experimental results in Different Prior Cognitive Level section, it can be seen that when the subjects have different knowledge reserves, the wavelet amplitude and WPCO of functional connectivity show significant differences. The hypotheses **H2a** and **H2b** cannot be rejected, i.e., the brain network is significantly different due to the different prior cognitive levels while engineering problem-solving.

Engineers with high prior cognitive levels hold more knowledge reserves and exploited more knowledge they have memorized. Thus, the wavelet amplitude increases significantly due to the activation of brain regions (Arun et al., 2020). Oppositely, engineers with low prior cognitive levels hold sketchy knowledge reserves (Hochberger et al., 2018). They cannot accurately mobilize prior knowledge to solve the engineering problems in the new design situation. Few brain regions participate in knowledge transfer and design activities, and the neural activation of brain regions descends.

The theory of cognitive structure held that learning a new paradigm is underlying the prior cognitive structure (Ausubel et al., 1968). An integrated cognitive structure (prior cognitive level) can effectively facilitate knowledge transfer. Therefore, we generally perceive that engineers with high prior cognitive levels own high stability, availability, and discriminability of cognitive structure. The intact cognitive structure improved their knowledge reserve of engineers. A previous fNIRS study showed that the brain synchronization of the PFC was greatly affected by the cognitive level in subjects with mild cognitive impairment (Udina et al., 2020). The cognitive level caused the interaction from the myogenic activity of smooth muscle and sympathetic/parasympathetic nervous systems, furthermore resulting in the decreasing coordination and synchronization of functional connectivity (Marusak et al., 2017).

Moreover, self-directed learning willingness is also a cognitive factor affecting knowledge transfer through behavioral measurement in psychology and management (Li et al., 2019). Engineers with high prior cognitive levels usually retain learning initiative and creativity. It is possible to construct new knowledge networks and switch suitable learning methods in the brain simultaneously. The complexity of their FC is significantly higher than those subjects with lower prior cognitive levels.

### Assessing the Effectiveness of Functional Connectivity in Knowledge Transfer

Related works corroborated that transfer distance and prior cognitive level significantly impact transfer performance using behavioral measurement (Li et al., 2019; Wang et al., 2021). In this study, the FC method based on fNIRS also confirmed the above views. Whether the statistical results of FC are significantly consistent with the results of behavioral measurement, this matter determines the effectiveness of the our proposal. According to the Pearson correlation analysis in Pearson Correlation Analysis Between the Performance of Knowledge Transfer and Functional Connectivity section, along with the increase of the knowledge transfer performance, the neural activity of **LOFA** and **LPTBA** also increases significantly. It indicates that the wavelet amplitude of FC is consistent with the behavioral measurement.

Meanwhile, the WPCO of **LIPFG-PIPFG**, **LPTBA-RIPFG**, **LFOA-LIPF**G were positively correlated with transfer performance. It shows that the multiple brain regions of the engineers cooperate to achieve knowledge transfer and design decisions. It is noteworthy that the connections in functional brain networks are not all beneficial to engineering design during engineering problem-solving. There are some connectivities, **LFOA-RPTBA** and **RFOA-LDLPFC**, whose WPCO are negatively correlated with transfer performance. Wrong association learning, incorrect analogy learning, set patterns of thinking, and distraction can produce this negative correlation, which will decrease the performance of knowledge transfer and the quality of the final design task (Macpherson and Stanovich, 2007). Therefore, the WPCO of FC is also consistent with the behavioral measurement.

The analysis above denotes that hypotheses **H3a** and **H3b** cannot be rejected. The results testify that brain FC is an effective method to evaluate the neurocognitive underpinnings of knowledge transfer. These two indicators are wavelet amplitude and WPCO. Note that only some brain regions and connections are significantly correlated with transfer performance, as shown in Figure 10, and the others are involved in knowledge transfer is unknown. Furthermore, complex cognitive processing is usually affected by the task situation, called context-dependent. Brain regions and functional connectivity in cognitive processing may be inconsistent in different situations, such as engineering design and art design (Vieira et al., 2020). Therefore, the FC of knowledge transfer in different design contexts can be further explored in future studies.

### About the Dorsolateral Prefrontal Cortex (DLPFC)

The experimental results of the wavelet amplitude, WPCO, and network complexity were analyzed. It was seen that LDLPFC and RDLPFC show different levels of activation and network connectivity, affected by either transfer distance or prior cognitive level, especially RDLPFC. Three main reasons are as follows:

- The M-WCST is more sensitive in detecting the prefrontal cortex, especially the DLPFC (Ni et al., 2017). The PFC is concerned in this study, and the concept-sorting tasks meet our requirements. The results also verify DLPFC is a crucial cortex in the functional connectivity of knowledge transfer.
- The DLPFC is associated with some brain functions, such as working memory, learning, decision, attention, and motivation (Boschin et al., 2017; Nakamura et al., 2020). An fMRI study showed that the changes in working memory tests were responsible for the different activation of the left and right PFC (Bomyea et al., 2018; Barbosa et al., 2020). In this study, the design situations of gearbox and gear pump involve these brain functions, which become necessary conditions for triggering the activation of DLPFC. The strong activation and functional connectivity in RDLPFC may be related to the distinctive functions of the right brain, such as intuition, space, and imagination (Tozzi et al., 2021).

### About the Orbitofrontal Area (OFA)

According to the above analysis, we can find that OFA plays an essential role in knowledge transfer while engineering problem-solving. It may be related to the distinctive functions of OFA, such as behavior decision, behavior control, behavior inhibition (Rolls, 2019).

- Behavior decision: Modality-independent decision-making is underlying the accumulation of sensory evidence and the duration of stimuli, mastermind by the frontal lobe network (de Winkel et al., 2017; Ma et al., 2021). In the learning task of knowledge transfer, the subjects decided on the beneficial contents from many stimulus materials. In the concept-sorting test, the subjects divided the concepts into two categories: “related-intention” and “unrelated-intention.”
- Behavior inhibitory control: A combined EEG-fNIRS study elaborated that the effect of fitness on inhibitory control was mediated by conflict monitoring. Additionally, the brain circuitry of inhibitory control sometimes improve concentration and gain higher rewards (Becker et al., 2017; Ludyga et al., 2019). The subjects avoided their unnecessary body movements to promote their concentration on the learning task. In the concept-sorting test, the subjects read the task situation according to the guidance text of the screen, and they need to inhibit irrelevant content and set patterns of thinking. In the formal test period, the subjects operated the mouse to classify concepts and exclude interference options.

### Limitations

First, due to the narrow scope of the investigation, the recruited subjects in this research were college students and young scholars. The sampling bias may impact the conclusions regarding knowledge transfer, and a future study will be carried out in the actual industrial situation. However, the difficulties of the design tasks in this study are entry-level. College students and young scholars have enough knowledge reserves to finish the experiment. Hence the sampling bias is within an acceptable range. Second, the learning willingness of the subjects affects the brain function connection. We did not discuss the specific correlation between the brain function networks and the six facets (efficiency, emotion, motivation, initiative, individuality, and creativity) of the self-directed learning readiness scale (SDLRS) (Hoban et al., 2005). Third, due to the physiological noise in fNIRS, Mayer waves may disturb the statistical results of the functional connectivity (Izzetoglu and Holtzer, 2020). To avoid the obstacle of single equipment on neurophysiological data acquisition, we will develop multimodal experiments with EEG-fNIRS to further explore the brain functional network of knowledge transfer.

## Conclusion

In this study, the neurocognitive mechanism of knowledge transfer while engineering problem-solving was evaluated by the functional connectivity. The results from 31 subjects showed that transfer distance, prior cognitive level, and transfer performance impacted the wavelet amplitude and WPCO. Concretely, the transfer distance and prior cognitive level significantly positively correlated to FC. Afterward, a significant correlation was observed between transfer performance and functional connectivity by a Pearson correlation analysis. Therefore, FC is an available method to assess the neurophysiological substrates of knowledge transfer. The DLPFC and OFA made many contributions to knowledge transfer while engineering problem-solving, owed to the specific brain functions or features of M-WCST. Statistical results demonstrated that the knowledge transfer procedure involves both the activation and inhibition of brain interconnectedness.

## Data Availability Statement

The original contributions presented in the study are included in the article/supplementary material, further inquiries can be directed to the corresponding author/s.

## Ethics Statement

The studies involving human participants were reviewed and approved by the Institutional Review Board (IRB) of Shanghai Jiao Tong University. The patients/participants provided their written informed consent to participate in this study. Written informed consent was obtained from the individual(s) for the publication of any potentially identifiable images or data included in this article.

## Author Contributions

XL and LB designed the study and edited the manuscript. FW designed the study, analyzed the data, and drafted the manuscript. YJ performed the experiment and statistical analysis. ZJ administrated this project. All authors contributed to the article and approved the submitted version.

## Funding

This research was supported by the National Natural Science Foundation of China (No. 71671113).

## Conflict of Interest

The authors declare that the research was conducted in the absence of any commercial or financial relationships that could be construed as a potential conflict of interest.

## Publisher's Note

All claims expressed in this article are solely those of the authors and do not necessarily represent those of their affiliated organizations, or those of the publisher, the editors and the reviewers. Any product that may be evaluated in this article, or claim that may be made by its manufacturer, is not guaranteed or endorsed by the publisher.
